# Bufei Yishen formula protects the airway epithelial barrier and ameliorates COPD by enhancing autophagy through the Sirt1/AMPK/Foxo3 signaling pathway

**DOI:** 10.1186/s13020-024-00905-1

**Published:** 2024-02-28

**Authors:** Lidan Jia, Xuefang Liu, Xinguang Liu, Qingzhou Guan, Yange Tian, Jiansheng Li, Peng Zhao

**Affiliations:** 1 Henan Key Laboratory of Chinese Medicine for Respiratory Disease, Henan University of Chinese Medicine, Zhengzhou, 450046 Henan Province China; 2 Collaborative Innovation Center for Chinese Medicine and Respiratory Diseases Co-Constructed by Henan Province & Education Ministry of P. R. China, Zhengzhou, 450046 Henan Province China; 3 Academy of Chinese Medical Sciences, Henan University of Chinese Medicine, Zhengzhou, 450000 China; 4 Department of Respiratory Diseases, The First Affiliated Hospital of Henan University of Chinese Medicine, Zhengzhou, 450000 China

**Keywords:** Chronic obstructive pulmonary disease, Bufei Yishen formula, Airway epithelial barrier, SIRT1/AMPK/FOXO3 signals, Autophagy

## Abstract

**Object:**

Bufei Yishen formula (BYF), a traditional Chinese medicine alleviates COPD symptoms and suppresses airway epithelial inflammation. In this study, we determined whether BYF protects the airway epithelial barrier from destruction in COPD rats.

**Methods:**

The protective effects of BYF on the airway epithelial barrier were examined in a rat COPD model. BEAS-2B epithelial cells were exposed to cigarette smoke extract (CSE) to determine the effect of BYF on epithelial barrier function. Transcriptomic and network analyses were conducted to identify the protective mechanisms.

**Results:**

Oral BYF reduced the severity of COPD in rats by suppressing the decline in lung function, pathological changes, inflammation, and protected airway epithelial barrier function by upregulating apical junction proteins, including occludin (OCLN), zonula occludens (ZO)-1, and E-cadherin (E-cad). BYF treatment reduced epithelial permeability, and increased TEER as well as the apical junction proteins, OCLN, ZO-1, and E-cad in BEAS-2B cells exposed to CSE. Furthermore, 58 compounds identified in BYF were used to predict 421 potential targets. In addition, the expression of 572 differentially expressed genes (DEGs) was identified in CSE-exposed BEAS-2B cells. A network analysis of the 421 targets and 572 DEGs revealed that BYF regulates multiple pathways, of which the Sirt1, AMPK, Foxo3, and autophagy pathways may be the most important with respect to protective mechanisms. Moreover, in vitro experiments confirmed that nobiletin, one of the active compounds in BYF, increased apical junction protein levels, including OCLN, ZO-1, and E-cad. It also increased LC3B and phosphorylated AMPK levels and decreased the phosphorylation of FoxO3a.

**Conclusions:**

BYF protects the airway epithelial barrier in COPD by enhancing autophagy through regulation of the SIRT1/AMPK/FOXO3 signaling pathway.

## Introduction

Chronic obstructive pulmonary disease (COPD) is a major cause of morbidity and mortality worldwide. It is characterized by progressive respiratory symptoms and airflow restriction. Exogenous noxious stimuli, such as cigarette and tobacco smoke as well as dust particles, exasperate the symptoms of COPD, because of their direct contact with the airway epithelium. This results in an aberrant immune and inflammatory response in the airways [16, 35]. Bronchodilators, including beta 2-agonists and anti-muscarinic drugs as well as corticosteroids, are the primary agents used for COPD treatment [28], however, they exhibit minimal efficacy and cause significant side effects, such as steroid insensitivity and a higher risk of pneumonia [6, 22]. Thus, effective and safe treatments for COPD are urgently needed.

The airway epithelial barrier, which is primarily maintained by tight junctions (TJs) and adhesive junctions (AJs), is important for maintaining airway integrity and defending against harmful substances [4, 7, 14]. Long-term cigarette smoke and other harmful gas exposure contribute to the breakdown of the epithelial barrier, resulting in chronic inflammation, which is the hallmark feature of COPD. Cigarette smoke decreases the expression of apical junction genes and proteins, including occludin (OCLN), zonula occludens (ZO)-1, and E-cadherin (E-cad), in the airway epithelium of COPD patients. This increases the permeability of the epithelium, which leads to lung damage induced by inhaled particles and infection [8, 13, 23, 27]. In addition, cigarette smoke increases EGF levels and activates EGFR, which is expressed in bronchial and alveolar epithelial cells. This results in the activation of extracellular regulated protein kinase (ERK) and upregulates IL-6 expression, which is associated with barrier function [24]. Furthermore, the ERK1/2 inhibitor U0126 and EGFR inhibitor AG1478 significantly inhibit TJ dissociation and DNA damage [32]. Therefore, protecting airway epithelial barrier function is a rational approach to attenuate airway diseases, such as COPD.

Traditional Chinese medicine (TCM) is widely used for the treatment of COPD because it exhibits good efficacy and low adverse effects. Bufei Yishen formula (BYF) (patent: ZL.201110117578.1) consists of multiple Chinese herbs and is specifically administered to COPD patients who have lung-kidney qi deficit syndrome [38]. Recent studies have indicated that BYF can ameliorate clinical symptoms in COPD patients, slow the decline of lung function, and enhance exercise tolerance [20]. Furthermore, oral BYF improves lung function, and inhibits systemic and local inflammation, and the protease/antiprotease imbalance in COPD rats [21]. However, the protective effect of BYF on the airway epithelial barrier during COPD remains unclear.

In the present study, we examined the effect of BYF on the airway epithelial barrier in COPD rats and cigarette smoke-induced airway epithelial cells. In addition, network analysis and transcriptomics were used to identify the underlying mechanisms and the active compounds in BYF that protect the epithelium barrier in vitro and in vivo.

## Materials and methods

### Chemicals and reagents

Hongqi Canal® filter cigarettes (tar 10 mg; nicotine concentration 1.0 mg; carbon monoxide 12 mg) were supplied by Henan Tobacco Industry Co., Ltd., (Zhengzhou, China). *Klebsiella pneumoniae* (strain: 46,114) was purchased from the National Medical Culture Center (Beijing, China) and administered to animals by preparing a physiological saline solution of 6 × 10^8^ colony forming units (CFU) per milliliter (mL). Bufei Yishen formula (composed of Ginseng Radix et Rhizoma, Astragali Radix, Lycii Fructus, etc.) was provided by the First Affiliated Hospital, Henan University of Chinese Medicine (Zhengzhou, China). Doxophylline (Doxo) tablets were obtained from Heilongjiang Fuhe Pharmaceutical Group Co. LTD (Heilongjiang, China). Nobiletin was obtained from Must Bio-Technology Co., LTD, (Chengdu, China).

The bicinchoninic acid (BCA) protein assay kit was purchased from Thermo Fisher Scientific (USA). Antibodies against ZO-1, ZO-2, occludin, E-cadherin, ERK, and phosphorylated (P)-ERK1/2 were purchased from Proteintech (Wuhan, China). Antibodies against EGFR, p-AMPKα, p-FOXO3a, and p-EGFR were purchased from Cell Signaling Technology (Shanghai, China). Antibodies against SIRT1 and LC3B were purchased from Gene Tex (Shenzhen China).

### Cell culture and treatment

Bronchial airway epithelial cells (BEAS-2B) were obtained from Procell (Wuhan, China) and cultured in DMEM (Solarbio) containing 10% serum in a humidified 5% CO_2_ incubator at 37 °C. The cells were grown in media devoid of serum for 3 h prior to treatment.

### Transepithelial electrical resistance (TER) and FITC-dextran permeability assay

BEAS-2B cells were seeded into a chamber. Then, TER was measured using an Epithelial Volt Ohm Meter (EVOM, World Precision Instruments, USA). Each well was measured three times in different directions and then averaged. The values are expressed as ohm (Ω) ⋅ cm^2^.

BEAS-2B cells were cultured in Transwell chambers and treated with drug and/or CSE after forming a monolayer. Then, 4 kDa FITC-glucan was diluted in PBS and added to the upper chamber. The fluorescence levels in the culture medium were measured using a SynergyHTX multifunctional microplate reader. Fluorescein concentrations were measured by comparison with a standard curve and the following formula was applied: Fluorescence permeability = The amount of FD4 in the lower chamber/the amount added.

### BYF preparation

The composition of BYF is listed in Table 1. To prepare BYF extract, Astragali Radix, Lycii Fructus, Epimedii Herba, Paeoniae Rubra Radix, and Pheretima were decocted twice with water. Next, Ginseng Radix Et Rhizoma, Ardisiae Japonicae Herba, Corni Fructus, Fritillariae Thunbergii Bulbus, Perillae Fructus, Schisandrae Chinensis Fructus, and Citri Reticulatae Pericarpium were extracted twice with 70% ethanol. The water and ethanol extracts were incubated overnight, then a dry powder was prepared after the supernatant was collected. There were 2.59 g of raw herbs contained in 1 g of dry powder. 0.22 g Powder was Dissolve in 1 ml double steaming water. COPD rats were administered the powder orally (10 ml/kg). In a previous study, UHPLC Q-Extractive Orbitrap-MS/MS was used to characterize the chemical constituents of the BYF extract [36].

**Table 1:** The composition of the Bufei Yishen formula

No.	Herbal drug	Latin scientific name	Plant part(s)	Amount (g)
1	Ginseng Radix et Rhizoma	*Panax ginseng* C.A Mey	Radix et Rhizoma	9
2	Astragali Radix	*Astragalus tibetanus* Bunge	Radix	15
3	Corni Fructus	*Cornus officinalis* Siebold & Zucc	Fructus	12
4	Lycii Fructus	*Lycium barbarum* L	Fructus	9
5	Schisandrae Chinensis Fructus	*Schisandra* *arisanensis* Hayata	Fructus	9
6	Fritillariae Thunbergii Bulbus	*Fritillaria thunbergii* Miq	Bulbus	9
7	Perillae Fructus	*Perilla frutescens *(*L.*) Britton	Fructus	9
8	Citri Reticulatae Pericarpium	*Citrus sinensis *(*L.*) Osbeck	Pericarpium	9
9	Epimedii Folium	*Epimedium* *acuminatum* Franch	Folium	9
10	Paeoniae Rubra Radix	*Paeonia anomala* L	Radix	9
11	Pheretima	*Pheretima aspergillum* (E. Perrier)		12
12	Ardisiae Japonicae Herba	*Ardisia japonica *(Thumb.) Blume	Herba	15

The BYF dry extract was added to a DM101 macroporous resin column after dissolving in double-distilled water. The product was subject to vacuum freeze-drying. The resulting powder was dissolved in dimethyl sulfoxide (DMSO) at 100 mg/ml and stored at − 20 °C for further cellular experiments and to identify the chemical constituents.

### COPD rats and drug administration

Sprague–Dawley rats, weighing 220 ± 20 g were obtained from the Vital River Laboratory Animal Technology Co., Ltd (Beijing China). The Animal Ethics Committee of Henan University of Traditional Chinese Medicine approved the animal protocols (DWLL202003210). The rats were randomly divided into four groups: control group, model group, BYF high- and low-dose groups, and Doxo group. Except for the control group, the other groups were exposed to cigarette smoke combined with *Klebsiella pneumoniae* to establish a COPD rat model from weeks 1–8. For weeks 9–16, the rats in the control and model group received normal saline by gavage, whereas the treatment groups were administered BYF (Dose of raw herbs:11.6 g/kg/d, 5.8 g/kg/d; Dose of BYF dry extract: 4.5 g/kg/d, 2.2 g/kg/d; 2.59 g of raw herbs contained in 1 g of BYF dry extract; 10 mL/kg) or Doxo (36 mg/kg/d). At end of week 16, the rats were sacrificed and the lung tissues were harvested. The left lung was fixed in paraformaldehyde and the others were stored at − 80 °C after quick-freezing in liquid nitrogen.

### Pulmonary function

From week 0–16, Tidal Volume (TV), Minute Volume (MV), Peak expiratory flow (PEF) and 50% expiratory flow (EF50) of the rats were measured using a whole-body plethysmograph (Buxco, FinePointe WBP, USA) every four weeks.

### Pathological changes

The left lung of the rats was perfused and fixed with 10% paraformaldehyde. Hematoxylin and eosin (H&E) staining was done to determine the overall structural changes in the lung. The airway and alveoli were observed under an optical microscope. The small airway wall thickness was measured using CaseViewer software. The CaseViewer software was used to take pictures of the small airway and measure the small airway wall thickness.

### Immunohistochemistry

After paraffin embedding, lung tissue sections (4 μm) were prepared and incubated overnight at 4 °C with primary anti-IL-1β, IL-6, TNF-α, SOD2, matrix metalloprotein (MMP)-2, and MMP-9 antibodies based on a routine immunohistochemical procedure. A secondary antibody was added and neutral gum was used to seal the sample. The resulting signal was observed using an optical microscope, photographed, recorded, and analyzed by Image Pro-plus 6.0 software for integrated optical density (IOD).

### Immunofluorescence (IF) staining

For staining, lung tissues and BEAS-2B cells were permeabilized, blocked, and fixed. The lung tissues and cells were probed with primary antibody for ZO-1 and occludin at 4 °C overnight. A fluorescence-conjugated secondary antibody (1:200) was incubated with the samples at room temperature away from light. Finally, random images were captured by fluorescence confocal microscopy and analyzed using Image J software.

### Western blot analysis

Protein was extracted from lung tissue and BEAS-2B cells using RIPA lysis buffer, separated by SDS-PAGE, and transferred to PVDF membranes. The membranes were blocked in 5% milk and incubated with specific primary antibodies. Goat anti-rabbit IgG was added and incubated at room temperature. After development, the resulting images were collected using an imaging system (Bio-Rad, USA), and Image Lab™ software was used to analyze the grayscale values of the target bands.

### Integrated analysis of network pharmacology and transcriptome data

The individual BYF compounds were identified by ultra-performance liquid chromatography/time-of-flight mass spectrometry (UPLC-Q/TOF–MS/MS). The target BYF compounds were predicted based on the PharmMapper databases (http://www.lilab-ecust.cn/pharmmapper). The component–target network of BYF was constructed using CytoScape v3.8.2 software.

RNA-seq technology was used to detect differentially expressed genes (DEGs) in BEAS-2B cells induced by CSE (Jingzhou Gene Technology Co., Ltd. Shanghai, China). The fold-change (FC) of the DEGs in the CSE group and the Control group was converted using the logarithm of base 2. Genes with log2(FC) C)2 or log2(FC) l 0.5 and a corrected Q value < 0.05 were considered differentially expressed.

Based on the String database (https://string-db.org/), a protein–protein interaction network (PPI) of the BYF targets and DEGs was constructed. According to the degree value, the top 50 core nodes were selected for the Kyoto Encyclopedia of Genes and Genomes (KEGG) pathway enrichment analysis and biological process analysis. The active components of BYF were analyzed by pathway-target-component correspondence and the representative components were selected for in vitro verification.

### Statistical analysis

Statistical analyses were done using SPSS 21.0 software. A one-way ANOVA was used for comparisons between measurement data groups. A pairwise comparison was performed with Dunnett^’^s T3 method for those with different variances. The significance level was α = 0.05 and the data are expressed as the mean ± SEM.

## Results

### BYF treatment ameliorates the severity of COPD in rats

**Fig. 1:**
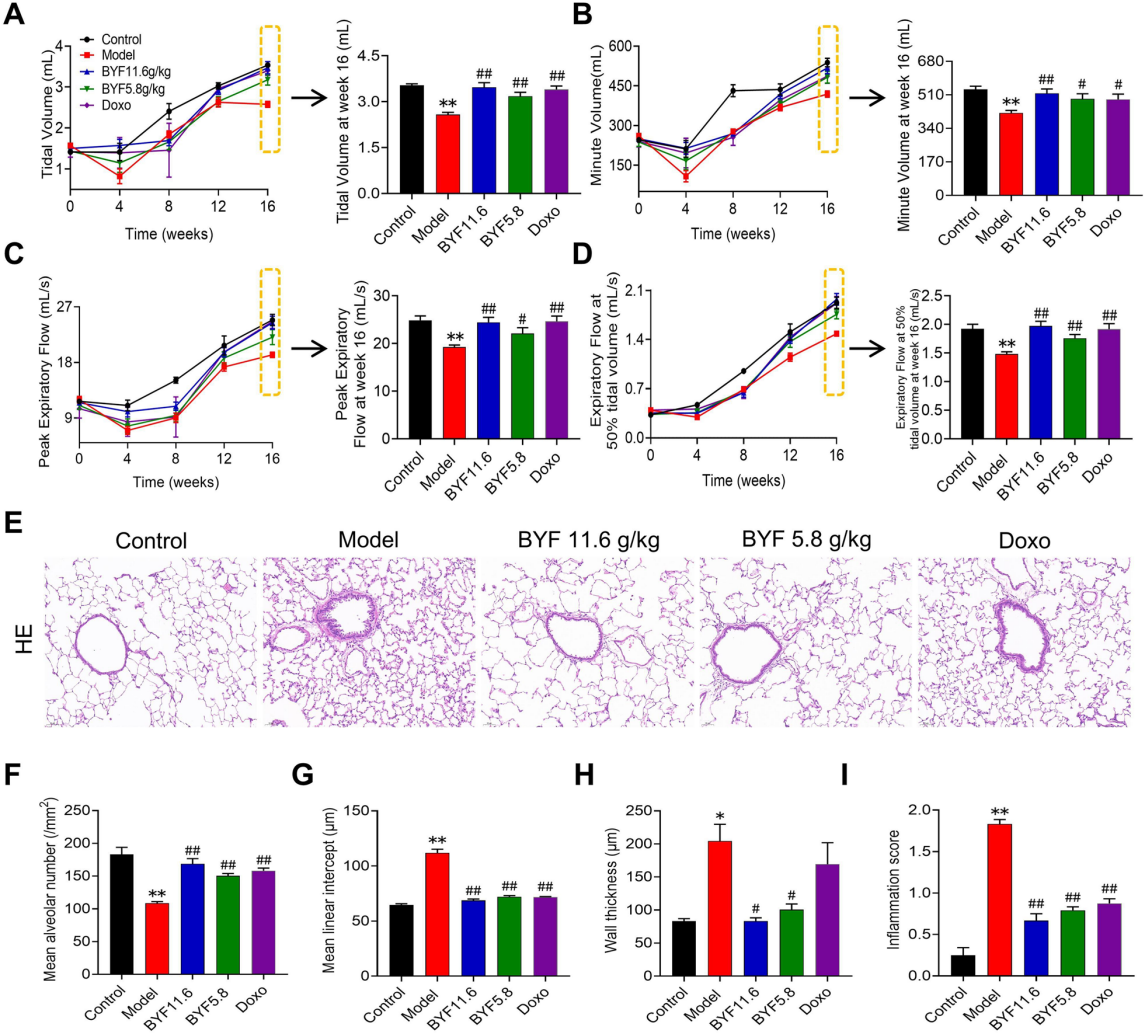
The Bufei Yishen formula improves CSE-induced pulmonary pathology and pulmonary function in COPD rats. BYF (11.6, 5.8 g/kg) and doxophylline (Doxo, 36 mg/kg) were administered to COPD rats for 9 to 16 weeks. **A** Tidal volume (TV). **B** Minute volume (MV). **C** Peak expiratory flow (PEF). **D** Expiratory flow at 50% tidal volume (EF50). **E** Pathological morphology of pulmonary tissue in each group (H&E, × 200). **F** Mean alveolar number (MAN). **G** Mean linear intercept (MLI). **H** Mean small airway wall thickness. **I** Inflammation score. Values are expressed as the mean ± SEM (n = 6), *P < 0.05, **P < 0.01 compared with the control group; ^#^*P* < 0.05, ^##^*P* < 0.01 compared with the model group

**Fig. 2:**
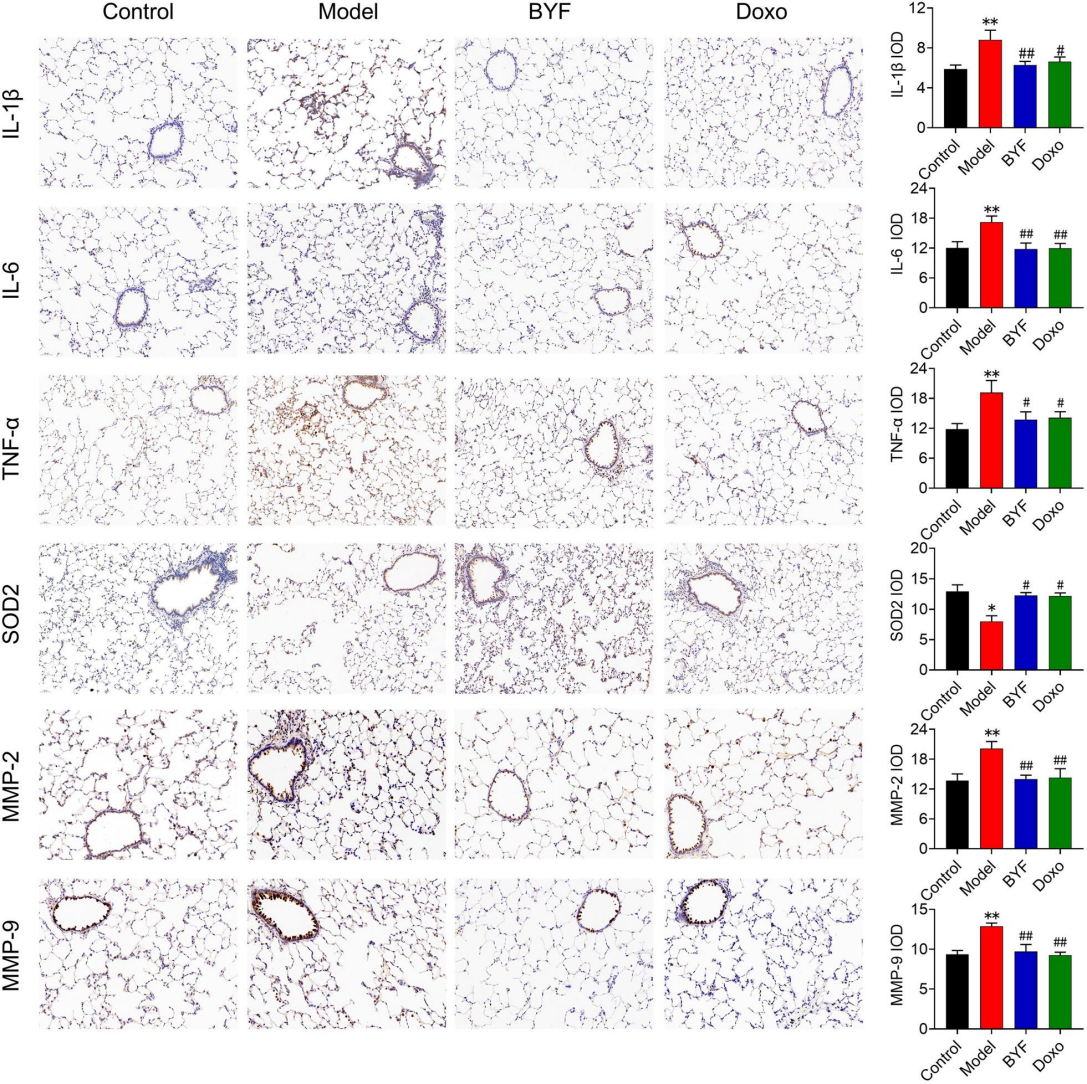
The Bufei Yishen formula ameliorates CSE-induced inflammation, oxidative stress, and protease expression in rats with COPD. BYF (11.6 g/kg) and doxophylline (Doxo: 36 mg/kg) were administered to COPD rats for 9–16 weeks. The expression of IL-1β, IL-6, TNF-α, SOD2, MMP-2, and MMP-9 in lung tissue (ession k their integral optical density (IOD). Data are expressed as the mean ± SEM (n = 6), *P < 0.05, **P < 0.01, versus the control group; ^#^*P* < 0.05, ^##^*P* < 0.01, versus the model group

To determine whether BYF can reduce the severity of COPD in animals, rats were exposed to cigarette smoke and bacterial infection for 8 weeks. The typical features of COPD, including lung function decline, lung damage, and the inflammatory response were evaluated. The results indicated that BYF treatment from weeks 9 to 16 significantly inhibited the decline of lung function, including TV, MV, PEF, and EF50, in COPD rats (Fig. 1A–D). H&E staining revealed that BYF treatment markedly suppressed pathological changes in the lung, such as alveolar structural damage, increased bronchiole wall thickness, and infiltration of inflammatory cells (Fig. 1E–J). In addition, inflammation, an imbalance of protease and antiprotease levels, and oxidative stress were central features of COPD. Thus, the effects of BYF on the expression of inflammatory cytokines, SOD2, and MMP in lung tissues were examined by immunohistochemistry. The results indicated that BYF treatment markedly decreased the expression of IL-1β, IL-6, TNF-α, MMP-2, and MMP-9, and increased SOD2 expression in the lung tissues of COPD rats (Fig. 2). These data suggest that BYF therapy attenuates the severity of COPD by enhancing lung function through a reduction of chronic lung damage and inflammation.

### BYF restores the airway epithelial barrier function in rats with COPD

**Fig. 3:**
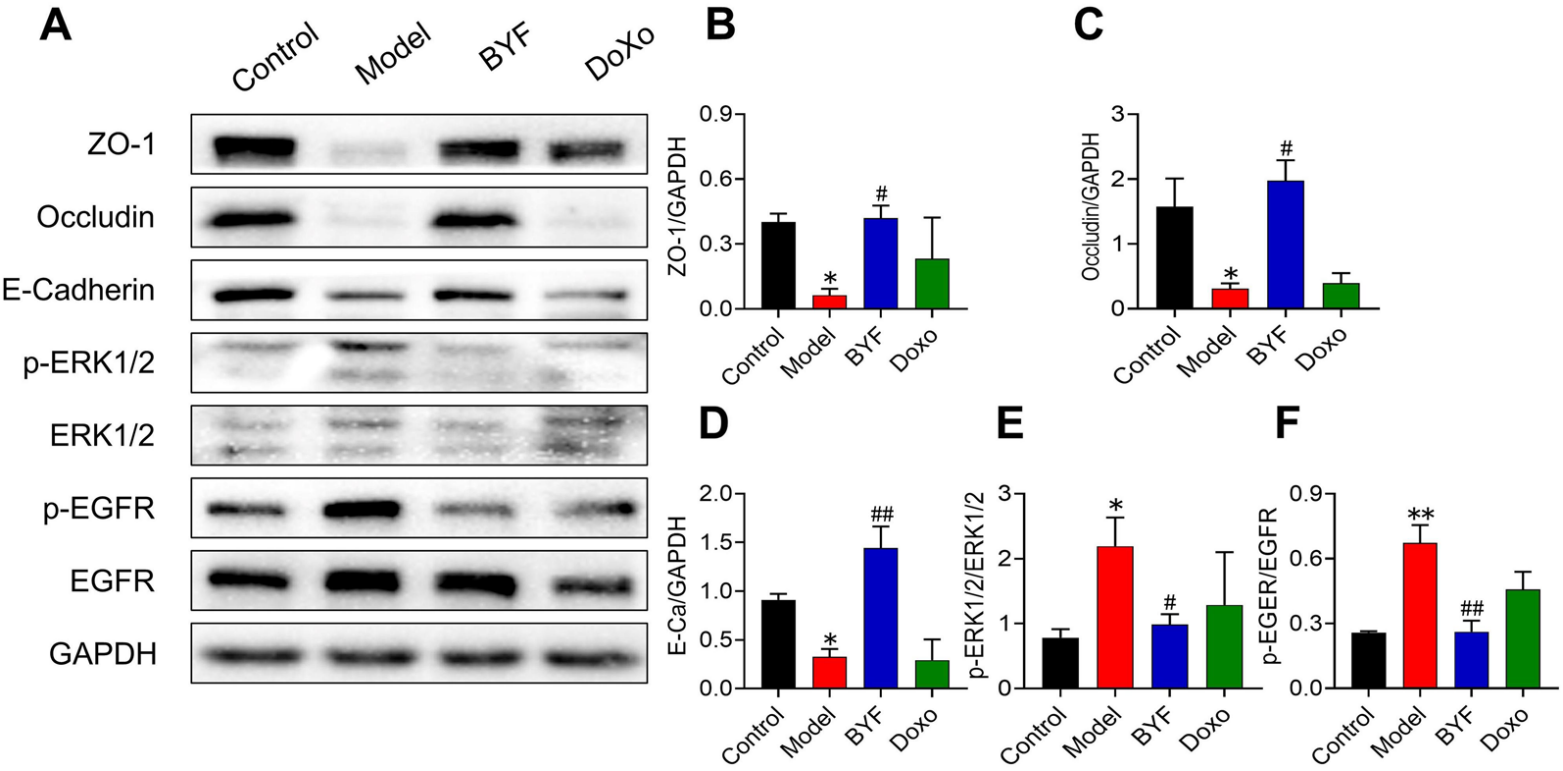
The Bufei Yishen formula increases the expression of the epithelial junction in COPD rats. BYF (11.6 g/kg) and doxophylline (Doxo: 36 mg/kg) were administered to COPD rats for 9 to 16 weeks. **A** The western bloting analysis of ZO-1, occludin, E-cadherin, p-ERK/1/2, and p-EGFR in the pulmonary tissue of COPD rats. **B**–**F** The quantitative analysis of the protein levels. Data are expressed as the mean ± SEM (n = 6), *P < 0.05, **P < 0.01, versus the control group; ^#^*P* < 0.05, ^##^*P* < 0.01, versus the model group

**Fig. 4:**
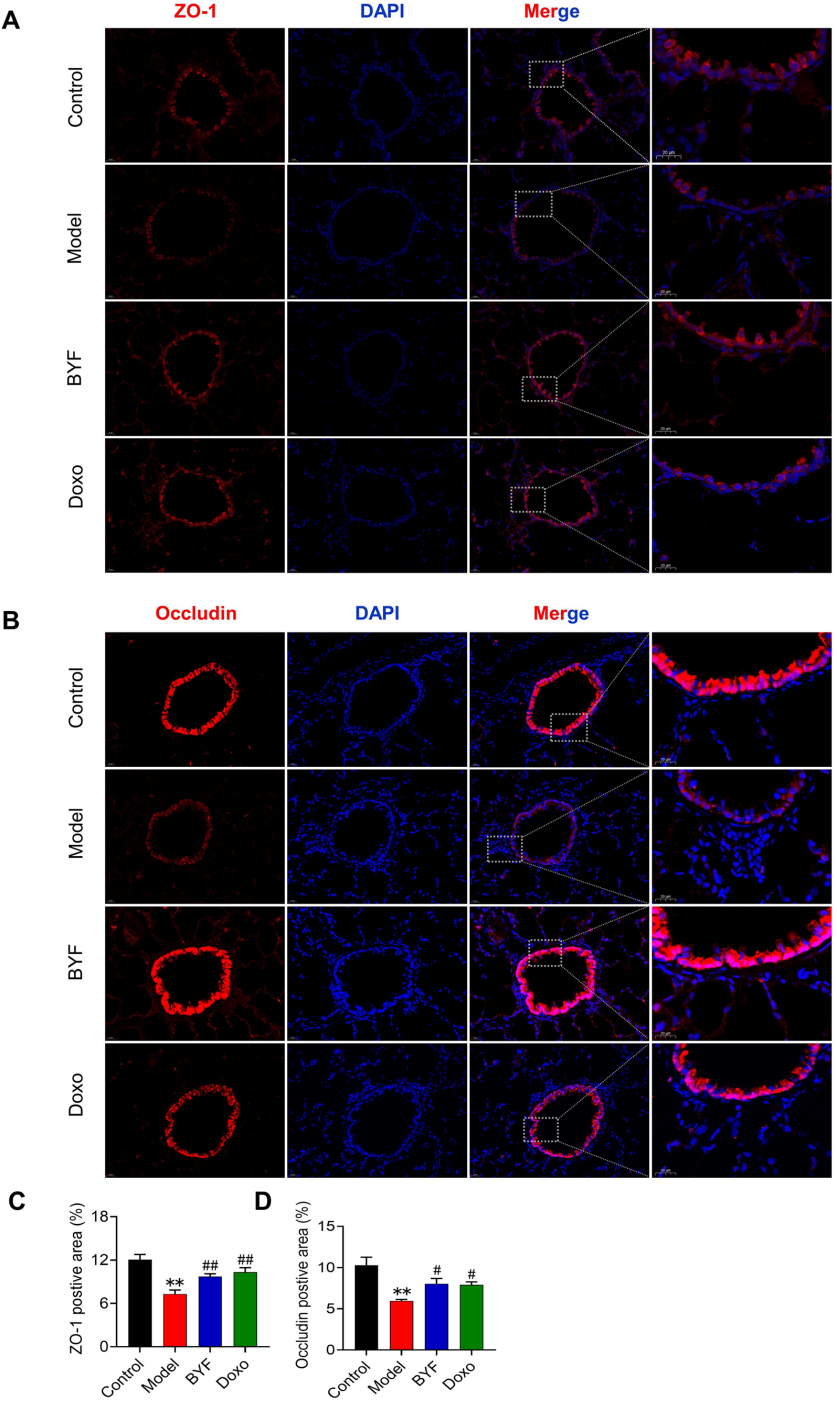
The Bufei Yishen formula enhances the integrity of tight junctions in COPD rats. BYF (11.6 g/kg) and doxophylline (Doxo: 36 mg/kg) were administered to COPD rats for 9 to 16 weeks. **A**, **B** The content of ZO-1 and OCLN in pulmonary tissues was determined using immunofluorescence. Fluorescence images (1 and OCLN in pulmonary tissue (**C**, **D**) Values are expressed as the mean ± SEM (n = 6), **P < 0.01, versus the control group; ^#^*P* < 0.05, ^##^*P* < 0.01, versus the model group

The airway epithelial barrier is the body’s first line of defense. It is a junction between epithelial cells, which can resist exogenous stimulation, including cigarette smoke and pathogenic infection, and plays an important role in the progression of chronic inflammation and acute exacerbation of COPD. We determined the effect of BYF treatment on the airway epithelial barrier in lung tissues by measuring the expression of apical junction proteins. Western blot analysis indicated that BYF treatment markedly inhibited the reduction of ZO-1, OCLN, and E-cadherin protein levels in the lung tissues of COPD rats (Fig. 3A–C). In addition, EGFR/ERK1/2 signaling in pulmonary tissues of COPD rats was activated, but suppressed following BYF treatment (Fig. 3D, E). An immunofluorescence analysis confirmed that BYF treatment increased the expression of OCLN and ZO-1 (Fig. 4A–D). These data indicate that BYF treatment restores airway epithelial barrier function in COPD by inducing apical junction protein production through the inhibition of EGFR/ERK1/2 signaling.

### BYF protects barrier dysfunction in CSE-induced bronchial epithelial cells

**Fig. 5:**
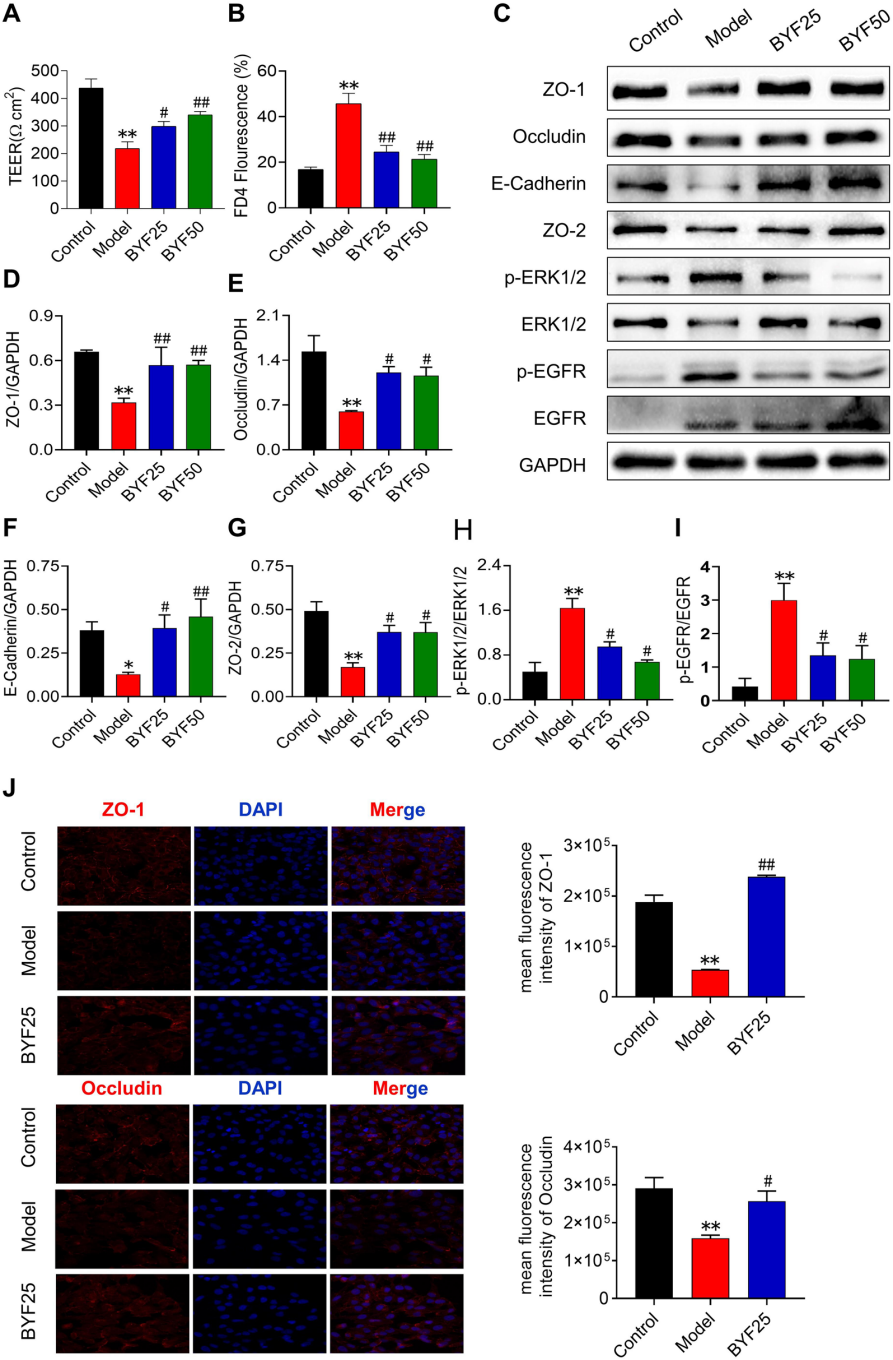
The Bufei Yishen formula (BYF: 25, 50 μg/ml) reduces paracellular permeability and increases the expression of epithelial junction in CSE-induced BEAS-2B cells. **A**, **B** TEER and permeability of BEAS-2B cell monolayers. **C**–**I** The levels of ZO-1, ZO-2, occludin, E-cadherin, p-ERK/1/2, and p-EGFR proteins in BEAS-2B cells. **J** The levels of ZO-1 and occludin proteins in BEAS-2B cells were detected by immunofluorescence. Data are expressed as the mean ± SEM (n = 3), *P < 0.05, **P < 0.01, versus the Control group; ^#^*P* < 0.05, ^##^*P* < 0.01, versus the Model group

To determine the effect of BYF on the airway epithelial barrier function, BEAS-2B cells were treated with BYF and CSE. A significant reduction in TER and an increase in FD4 permeability in BEAS-2B cells were observed following CSE exposure. These changes were suppressed in BYF-treated cells (Fig. 5A, B). In addition, the apical junction proteins, OCLN, ZO-1, E-cad, and ZO-2, in CSE-treated BEAS-2B cells were inhibited by BYF treatment (Fig. 5C–F). We also observed that BYF treatment suppressed the activation of EGFR/ERK1/2 signaling in BEAS-2B cells induced by CSE exposure (Fig. 5G, H). To determine the localization of epithelial junctional proteins, immunofluorescence was performed for OCLN and ZO-1. The results indicated that these apical junction proteins were decreased in CSE-exposed BEAS-2B cells, which was inhibited by BYF treatment (Fig. 5I). Our findings suggest that BYF directly attenuates CSE-induced epithelial barrier dysfunction by enhancing the expression of apical junction proteins.

### Transcriptomics and network analysis of the protective mechanisms of BYF

**Fig. 6:**
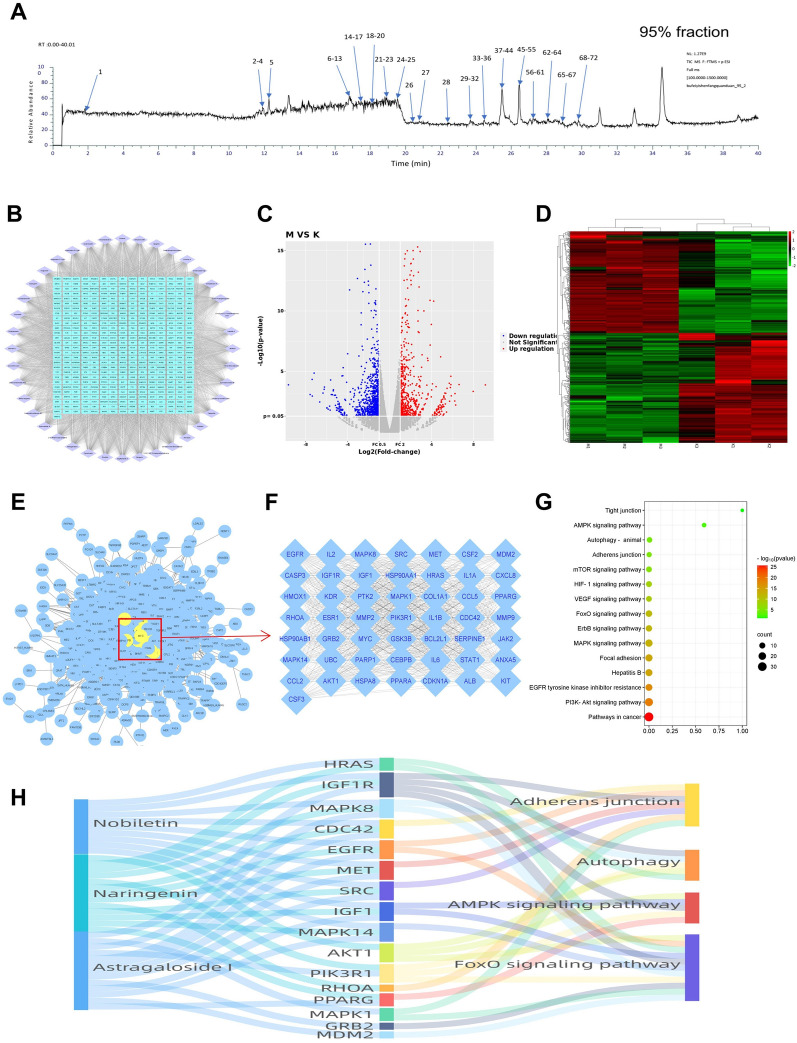
Comprehensive analysis of the targets of the Bufei Yishen formula and COPD genes. **A** UHPLC Q-Extractive Orbitrap-MS/MS was used to characterize the chemical constituents of the 95% fraction of BYF extract. **B** Compound and target network of the Bufei Yishen formula. **C** DEG volcano map of Model (BEAS-2B cell induced by CSE) vs. Control (BEAS-2B cell). **D** DEG cluster heatmap of Model (BEAS-2B cell induced by CSE) vs. Control (BEAS-2B cell). **E** The PPI network was constructed by combining Bufei Yishen formula targets and COPD genes. **F** The top 50 genes and targets were based on the degree ranking. **G** The top 50 targets and genes were analyzed using DAVID for KEGG pathway enrichment. **H** Sankey diagram of the components and targets associated with tight junctions, adherens junctions, autophagy, and the AMPK and FoxO signaling pathways

To identify the molecular mechanisms of the barrier protective effect of BYF, the compounds involved in BYF and their targets as well as the differentially expressed genes (DEGs) in CSE-exposed BEAS-2B cells were analyzed. First, 58 BYF compounds were identified using UPLC-Q/TOF–MS/MS (Table 2) and 421 associated targets were predicted (Fig. 6A). RNA-seq analysis revealed 572 DEGs in CSE-exposed BEAS-2B cells, including 275 upregulated and 297 downregulated genes (Fig. 6B). Finally, 917 target proteins together with 572 DEGs were used to establish a PPI, and the top 50 core nodes with a degree value ≥ 73 used for a KEGG pathway enrichment analysis. As shown in Fig. 6E, multiple pathways, including the AMP-activated protein kinase (AMPK) signaling pathway, Forkhead box O signaling pathway, EGFR tyrosine kinase inhibitor resistance, focal adhesion, and adherens junction, were associated with the barrier protective effects of BYF. The compounds, target proteins, and signaling pathways were also used to construct a Sankey diagram (Fig. 6F). These data suggest that BYF contains a variety of pharmacological compounds that target diverse proteins with multiple bioactive functions. They may represent the potential mechanism of the active substances of BYF that protect barrier function.

**Table 2:** The main compound of 95% fraction of BYF

No.	Compound	RT	m/z (expected)	m/z (delta) (ppm)
1	*p*-Hydroxy-cinnamic acid	1.97	165.0543	3.1797
2	Bergenin	11.23	329.088	0.0386
3	*cis*-Cryptochlorogenic acid/*trans*-cryptochlorogenic acid/chlorogenic acid	11.69	355.1045	− 2.6002
4	Quercetin-rha-tri-hex	11.82	935.2681	− 0.5580
5	Loganin	12	391.1616	− 2.7547
6	Apigenin-rha-glu	16.15	579.1729	0.3379
7	Kaempferol-3-*O*-gal	16.23	449.1095	− 0.2104
8	Hexandraside E	16.25	679.2266	N/A
9	Albiflorin	16.35	481.1723	− 0.7564
10	Rhoifolin	16.55	579.1729	0.3379
11	Hesperidin	16.79	611.1998	− 0.7513
12	Dihydroxy-trimethoxyflavone	16.81	345.0985	− 1.6491
13	Hesperetin-7-*O*-glu	16.81	465.1396	2.5973
14	Sibelicin glycoside	16.97	592.3843	2.2285
15	Diosmetin-6-C-glu	17.09	463.1247	− 1.1973
16	Diosmin	17.19	609.1825	1.1943
17	Peimisine	17.72	428.3171	0.9453
18	Des-*O*-Methylicariin/Epimedoside A	17.86	663.2299	1.5017
19	Meranzin/Isomeramazin (a)	18	261.1117	3.7183
20	Hesperetin-7-*O*-glu	18.3	465.1396	1.0227
21	Hesperetin	18.3	303.0875	− 1.2284
22	Zhebeininoside	19.03	594.4004	3.1674
23	Peimine A	19.17	432.3473	3.3651
24	Neoicariin/Wushanicariin/Icariside I	22.26	531.1852	4.9045
25	Isopeimine A	23.52	432.3473	3.2239
26	20(R)-Ginsenoside Rh1	23.69	639.4458	4.2965
27	Benzoylpaeoniflorin/Paeonivayin	24.5	585.1964	4.8168
28	Naringenin	24.51	273.075	1.0505
29	Apigenin	24.59	271.0593	4.6000
30	20(S)-Ginsenoside Rh1	24.81	639.4478	3.7459
31	Desmethylanhydroicaritin	25.01	355.1197	− 1.4033
32	Anhydroicaritin-3-*O*-Î′-l-Rhamnosyl-7-*O*-Î′-d-Glucopyranoside/Sagittatoside A	25.15	677.2426	4.2576
33	Benzoylpaeoniflorin/Paeonivayin	25.21	585.1951	4.5351
34	2′′-*O*-Rhamnosylicariside II/Anhydroicaritin 3-*O*-2′′-rha-rha	25.42	661.2476	4.9217
35	Isosinensetin	25.66	373.1275	4.6685
36	Astragaloside I/Isoastragaloside I	25.78	869.4894	1.9424
37	Hexamethoxyflavone (a)	25.86	403.1381	4.7494
38	Deacetylnomilin	25.92	473.2163	4.5324
39	Icariside II(Baohuside I)	26.02	515.1907	4.8065
40	Anhydroicaritin	26.02	369.1328	4.8289
41	Sinensetin	26.16	373.1279	4.0054
42	Gomisin L1	26.26	387.18	3.6856
43	Methyl eugenol	26.26	179.1067	2.8377
44	Tetramethyl-*O*-scutellarein/Tetramethyl-*O*-isoscutellarein/Tetramethoxyflavone (a)	26.28	343.1173	4.0303
45	d-ribo-Phytosphingosine	26.42	318.2998	4.5209
46	Rosmarinic acid methylester	26.57	375.1076	2.7794
47	Nobiletin	26.61	403.1384	4.2323
48	a-Linolenic acid	26.63	279.2308	2.6389
49	Tetramethyl-*O*-scutellarein/Tetramethyl-*O*-isoscutellarein/Tetramethoxyflavone (b)	26.83	343.1174	3.9167
50	3,5,6,7,8,3′,4′-Heptamethoxyflavone	26.97	433.1488	4.0950
51	Ginsenoside Rg5/Rk1	27.17	767.4932	4.7247
52	Schisandrol B/Epigomisin O	27.29	417.19	4.8338
53	Tangeretin	27.29	373.1277	4.2961
54	Epimedokoreanin B	28.52	423.179	4.2929
55	Benzoyl gomisin H	28.52	523.2314	3.9361
56	Schisantherin A	29.04	537.2109	1.5468
57	13-Hydroxy-9,11-octadecadienoic acid	29.59	297.2416	− 1.7196
58	Gomisin L1	29.71	387.18	3.7644

### The active compounds of BYF prevent barrier dysfunction and activate AMPK-FoxO3a-mediated autophagy

**Fig. 7:**
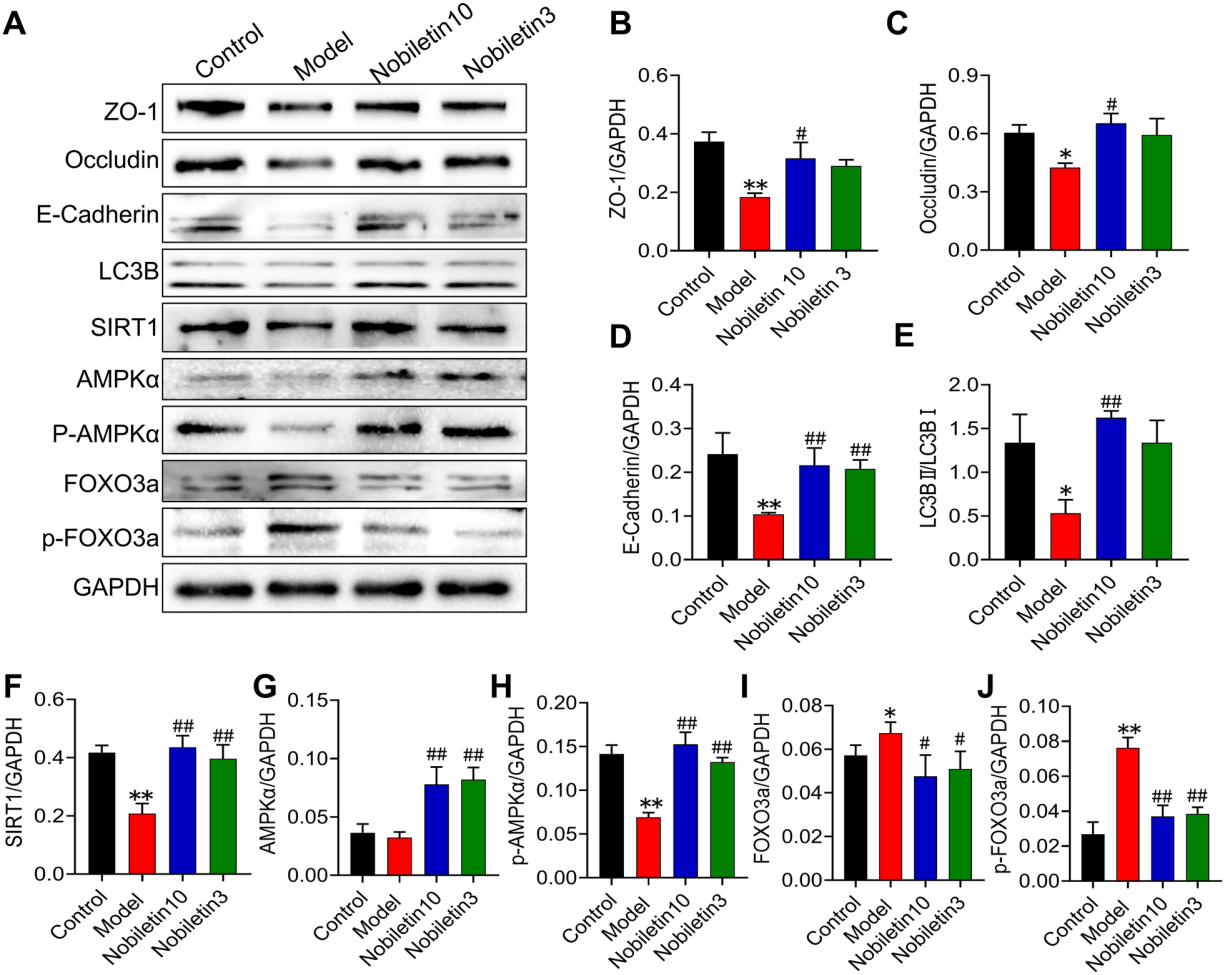
The active compounds of BYF prevent barrier dysfunction and activate SIRT1/AMPK/FoxO3-mediated autophagy in BEAS-2B cells exposed to CSE. **A**–**J** The expression of ZO-1, occludin, E-cadherin, LC3B, SIRT1, AMPK, p-AMPK, FOXO3a and p-FOXO3a in BEAS-2B cells treated with nobiletin (3, 10 μM), and the gray values were expressed as the target band /GAPDH. Data are expressed as the mean ± SEM (n = 3), *P < 0.05, **P < 0.01, versus the control group; ^#^*P* < 0.05, ^##^*P* < 0.01, versus the model group

The above analysis revealed that BYF contains multiple components and regulates various targets that are involved in regulating signaling networks. Moreover, previous studies have shown that the AMPK-Foxo3-autophagy pathway participates in epithelial barrier function [5, 19]. Network analysis also demonstrated that nobiletin, the active compound of BYF, regulates the Sirt1/AMPK-Foxo3 signaling pathway as well as autophagy. In vitro experiments demonstrated that nobiletin therapy could increase the levels of the apical junction proteins, including OCLN, ZO-1, and E-cad in CSE-exposed BEAS-2B cells (Fig. 7A–C). Furthermore, nobiletin significantly increased LC3B, sirtuin 1 (SIRT1), and phosphorylated AMPK protein levels, while decreasing FoxO3a phosphorylation (Fig. 7D–G). These data suggest that the active compound in BYF protects barrier function by enhancing autophagy through the regulation of AMPK-Foxo3a signals.

## Discussion

COPD is a serious threat to public health because of its high prevalence and significant economic burden [1, 37]. The drugs that are currently available for COPD, such as bronchodilators and glucocorticoids, have exhibited limited success. Thus, effective strategies are urgently needed. TCM formulas contain multiple medicinal herbs that are prescribed for COPD based on the TCM theory, which has proven to be effective and safe. Bufei Yishen formula, a TCM formula for COPD, reduces the exacerbation frequency and improves lung function and exercise capacity in COPD patients [18]. In the present study, BYF treatment improved pulmonary function, suppressed pathological changes, the inflammatory response, and protease levels, and protected airway epithelial barrier function by upregulating apical junction protein levels in the lung tissue of COPD rats. Moreover, BYF consistently inhibited the reduction of apical junction protein levels in CSE-induced BEAS-2B cells. We integrated the results of transcriptomic and network analyses to examine the protective mechanisms of BYF on barrier function and found that the regulation of Sirt1/AMPK/Foxo3 signals and autophagy may be associated with the protective effect of BYF.

The airway epithelium forms the first barrier against harmful substances, such as allergens, pollutants, and pathogens, which are the main risk factors for the progression of chronic lung disease [26, 30]. Studies show that damage to the airway epithelial barrier is an important characteristic of COPD [2]. For example, cigarette smoke can reduce the levels of apical junction proteins, such as OCLN, ZO-1, and E-cad, which lead to disruption of the airway epithelial barrier and subsequently facilitate viral and bacterial infection, immune cell recruitment, and sustained inflammation of the lung tissue [12, 15, 31]. Thus, maintaining the airway epithelial barrier integrity is an effective approach for treating COPD. In the present study, the levels of ZO-1, OCLN, and E-cad protein in the lung tissue of COPD rats were markedly decreased and inhibited by BYF treatment. In vitro experiments also demonstrated that BYF suppresses the reduction of OCLN, ZO-1, and E-cad, decreases TER, and increases FD4 permeability in BEAS-2B cells induced by CSE. These results indicate that BYF effectively protects airway epithelium barrier function, which may associated with the therapeutic effects of BYF.

To further explore the protective mechanism of BYF on epithelial barrier function, the integration of network pharmacological, transcriptomic, and in vitro experiments was applied to analyze the effective components of BYF, their targets, and associated biological function. First, we identified 58 compounds from BYF, and then predicted 421 targets for these compounds. RNA-seq provides valuable biological information by identifying differentially expressed genes, thereby identifying putative mechanisms of drug treatment and the pathogenesis of complex diseases. We used RNA-seq to identify DEGs in BEAS-2B cells induced by CSE. These DEGs were primarily associated with the Sirt1, AMPK, and Foxo3 pathways as well as autophagy, which may be involved in the protective mechanism of BYF.

Autophagy is a self-regulatory process that occurs during stress and starvation, which is important to the pathogenesis of COPD [3, 25, 34]. Studies have suggested that CS induces autophagy, which can accelerate the COPD process [33]. In addition, autophagy can enhance epithelial barrier function and reduce paracellular permeability by regulating epithelial junction proteins [25]. Abnormal autophagy induced by cigarette smoke is closely associated with COPD epithelial barrier dysfunction, which can lead to defects in airway epithelial cell connection and damage to the epithelial barrier [29]. As a component of the autophagosome membrane, LC3B is widely considered an autophagic activity marker [17]. In vitro experiments indicated that CSE reduced the expression of LC3B, ZO-1, occludin, and E-cadherin, which is suppressed by nobiletin, the active compound of BYF. Moreover, autophagy is regulated by SIRT1, AMPK, FOXO, and other signaling pathways [9–11]. These findings indicate that nobiletin treatment significantly upregulates SIRT1 and p-AMPKα and downregulates p-FOXO3a expression in a dose-dependent manner. They also show that nobiletin enhances autophagy by attenuating the SIRT1/AMPK signaling pathway.

In summary, our present data confirmed the protective effect of BYF on COPD rats by reversing the decrease in lung function, pathological changes, inflammation. Interestingly, our data also showed that BYF could directly protect airway epithelial barrier function through enhancing autophagy by regulating SIRT1/AMPK/FOXO3. TCM is characterized by “from the clinic to the laboratory”, and then “laboratory back to the clinic”. Thus, this study explored that BYF, the effective prescription for treating COPD, maintains airway epithelial barrier function in COPD by enhancing autophagy through the regulation of SIRT1/AMPK/FOXO3 signaling, which provide the basis for clinical application. Furthermore, the effect of the components identified from BYF, were needed to evaluated in COPD rats, while the synergistic effects of different compounds also need to be explored in the future. These results also provide candidate compounds for new drug development.

## Conclusion

In conclusion, BYF protects the airway epithelial barrier against COPD by ameliorating autophagy through the SIRT/AMPK/FOXO3 signaling pathway. We used a combination of network pharmacology and transcriptomics to provide new insight into the mechanism through which BYF ameliorates COPD by protecting the airway epithelial barrier.

## Data Availability

The datasets presented in this study can be found in online repositories. The names of the repository/repositories and accession number(s) can be found in the article/supplementary material.
